# The Impact of Maternal Body Composition and Dietary Fat Consumption upon Placental Lipid Processing and Offspring Metabolic Health

**DOI:** 10.3390/nu12103031

**Published:** 2020-10-03

**Authors:** Zachary J. W. Easton, Timothy R. H. Regnault

**Affiliations:** 1 Department of Physiology and Pharmacology, Western University, Medical Sciences Building Room 216, London, ON N6A 5C1, Canada; tim.regnault@uwo.ca; 2 Department of Obstetrics and Gynaecology, London Health Science Centre-Victoria Hospital, B2-401, London, ON N6H 5W9, Canada; 3 Children’s Health Research Institute, 800 Commissioners Road East, London, ON N6C 2V5, Canada; 4 Lawson Health Research Institute, 750 Base Line Rd E, London, ON N6C 2R5, Canada

**Keywords:** developmental origins of health and disease, gestational diet, maternal body composition, offspring metabolic health, placenta, lipid metabolism

## Abstract

The proportion of women of reproductive age who are overweight or obese is increasing globally. Gestational obesity is strongly associated in both human studies and animal models with early-onset development of adult-associated metabolic diseases including metabolic syndrome in the exposed offspring. However, animal model studies have suggested that gestational diet in obese pregnancies is an independent but underappreciated mediator of offspring risk for later life metabolic disease, and human diet consumption data have highlighted that many women do not follow nutritional guidelines prior to and during pregnancy. Thus, this review will highlight how maternal diet independent from maternal body composition impacts the risk for later-life metabolic disease in obesity-exposed offspring. A poor maternal diet, in combination with the obese metabolic state, are understood to facilitate pathological in utero programming, specifically through changes in lipid handling processes in the villous trophoblast layer of the placenta that promote an environment associated with the development of metabolic disease in the offspring. This review will additionally highlight how maternal obesity modulates villous trophoblast lipid processing functions including fatty acid transport, esterification and beta-oxidation. Further, this review will discuss how altering maternal gestational diet may ameliorate these functional changes in lipid metabolic processes in the obese placenta.

## 1. Introduction

Throughout the gestational period, maternal nutrient handling must adapt to the increasing needs of the growing fetal-placental unit to ensure developmental processes continue in a healthy and physiological manner. For example, maternal insulin sensitivity diminishes, and fasting serum lipid levels rise late in gestation to preserve necessary macronutrients for trans-placental transport into fetal circulation [1,2,3]. However, there is a fine balance within these physiological metabolic alterations that, when disrupted by environmental influences, can shift the course of in utero programming to promote the early life development of metabolic disorders in the offspring. Maternal gestational obesity is one such environmental influence that has been well associated with poor health outcomes in exposed offspring. Importantly, recent animal models have highlighted that, in addition to maternal obesity, a maternal diet high in fat is an important independent regulator of offspring lifelong metabolic health. Thus, this review will primarily discuss how maternal gestational dietary composition in obese pregnancies influences future offspring health independent from maternal body composition.

Furthermore, alterations in lipid processing functions of the placenta—including fatty acid (FA) transport, lipid esterification and FA beta-oxidation—have been thought to modulate materno-fetal lipid transport and the resulting changes to fetal lipid exposures may underlie metabolic disease programming. This review will additionally highlight how maternal obesity modulates these lipid handling processes in the placenta and discuss how maternal diet may program these placental processes independently from increased maternal adiposity.

## 2. Maternal Obesity and Offspring Metabolic Health

The study of the impacts of maternal gestational environment on fetal growth and development is encompassed within the field of research known as The Developmental Origins of Health and Disease (DOHaD) [4,5]. This field of study evolved from the observations of Anders Forsdahl and David Barker in the 1970s and 80s whereby Forsdahl originally described an increased risk of death by coronary heart disease in those who were relatively impoverished during childhood, but later experienced prosperity [6]. Barker expanded these observations to include gestational influences and reported that low birthweight babies were at a greater risk for developing metabolic complications such as obesity, type 2 diabetes mellitus (insulin resistance) and metabolic syndrome in adulthood [4,5]. This field of study has since expanded to include the observed increased risk of later life non-communicable diseases associated with metabolic syndrome in offspring born in an environment of maternal diet-induced obesity [7,8].

The World Health Organization (WHO) categorizes healthy bodyweight in both adults and children via body mass index (BMI, kg/m^2^), whereby a BMI > 25 is overweight and a BMI > 30 is obese [9]. The effects of an increased maternal body mass and associated adiposity during the gestational period on offspring later life health has been extensively documented in humans via population studies and meta-analyses [10,11,12,13,14,15]. In line with the DOHaD concept, obesity-exposed offspring have been found to be at a greater risk for later-life metabolic health issues due in part to an increased prevalence of having a birthweight that is not appropriate for their gestational age (AGA) [10,13]. While maternal gestational obesity has largely been associated with infants being born Large for their Gestational Age (LGA), there has also been a link between maternal obesity and greater risk of the offspring being born Small for their Gestational Age (SGA) [10,11,14]. Independent from maternal factors, LGA and SGA offspring are at an increased risk for developing non-communicable “adult-associated” metabolic disorders as early as four years of age [12,13]. Concerningly, however, there are reports that children born to obese women are more likely to develop metabolic disorders regardless of their birthweight, suggesting that maternal body composition during pregnancy influences offspring metabolic health simply beyond alterations in birthweight [14]. Indeed, recent studies have suggested that maternal factors including pre-pregnancy BMI may better predict the development of offspring health complications than birthweight alone [14,15].

The negative influence that maternal adiposity has on offspring metabolic health has additionally been reported in numerous animal models that attempt to elucidate the mechanisms that lead to early-life metabolic diseases in obesity-exposed offspring [16,17]. While maternal diet-induced obesity has been well associated with poor fetal metabolic outcomes in these models, it is important to note that variations are present in the dietary fat contents and periods of exposure used in these studies (Table 1). Rodent models in particular have been heavily utilized and the development of metabolic disorders in the offspring born to high-fat diet (HFD)-induced obese dams has been described the result of pathological in utero programming [18,19]. The high-fat-exposed rodent offspring have been found to exhibit an abnormal lipid profiles including hepatic steatosis that ultimately leads to Non-Alcoholic Fatty Liver Disease (NAFLD) and fibrosis at early life stages [20]. Altered glucose homeostasis is also prevalent in these obesity-exposed rodent offspring and is manifested as insulin resistance and an eventual development of type 2 diabetes mellitus (T2DM) during adolescence [21,22]. The altered glucose and liver lipid metabolism observed in these offspring has been thought to be a precursor to the ultimate development of metabolic syndrome in gestational obesity-exposed adolescents [23,24].

Larger mammal species, including sheep, have also been used to study maternal overfeeding and obesity and its subsequent effects on offspring health and disease. As observed in human meta-analyses and rodent experiments, sheep offspring exhibit metabolic dysfunction both neonatally and into adulthood —including increased prevalence of obesity and aberrant lipid and glucose metabolism—in response to maternal obesity during gestation [25,26,27]. Additionally, the non-human primate (NHP) model has been well utilized and describes dysregulated fetal hepatic lipid and glucose metabolism as an underlying pathology of maternal obesity mediated offspring metabolic disease development [28,29]. 

Together, these human meta-analyses and animal models demonstrate that maternal obesity during the gestational period primes the exposed offspring for dysregulated lipid and glucose metabolism that ultimately results in metabolic disease development early in life.

## 3. Is Maternal BMI an Accurate Predictor of Offspring Metabolic Health?

The reports from these human and animal studies that link maternal obesity to offspring metabolic disease are of increasing importance to healthcare systems as the prevalence of obesity worldwide has reached unprecedented rates over the last several decades [30]. The WHO estimates that about 40% of men and women over the age of 18 were overweight or obese in 2016, and that proportion continues to rise [30]. More specific to pregnancy outcomes and in line with data from most industrialized nations, Health Canada reported in 2012–13 that 24% of Canadian women between 20–39 years of age (child-bearing age) were obese, and 44% had a waist circumference that was predictive of high risk for the development of health complications [31]. These reports suggest that the prevalence of early-onset metabolic syndrome in offspring will only continue to increase alongside the rising rates of maternal obesity.

Recent animal models utilizing dietary interventions in obese pregnancies have highlighted that body composition metrics may not be the most accurate predictors of offspring future metabolic health and that maternal gestational diet is an important influence (Table 1). For example, in sheep models of gestational overfeeding-induced obesity a maternal dietary intervention early in gestation resulted in lowered circulating plasma triglyceride levels (improved lipid metabolic function) as well as decreased plasma insulin levels (improved glucose metabolism) in fetuses from obese pregnancies at both mid and late gestation [27]. Additionally, NHP data suggest that there are vast differences in the metabolic health of fetuses from obese mothers that consume different diets during gestation [28,29,32]. McCurdy et al. (2009) identified that a diet reversal to a control diet in obese pregnant Japanese macaques was sufficient to improve liver steatosis in third trimester fetuses, suggestive of a decreased risk of postnatal NAFLD. Subsequent studies described reductions in maternal and fetal dyslipidemia and oxidative stress in diet-reversed obese pregnancies leading to benefits in fetal liver development during the third trimester [32]. Additionally, improved third trimester pancreatic islet vascularization has been reported and highlights that these offspring would be less susceptible to later-life development of type 2 diabetes mellitus [29]. These NHP studies highlight that maternal gestational obesity alone may not best predict offspring metabolic health and suggest that gestational diet is important in determining metabolic health risk in the obesity-exposed offspring.

Rodent models of obese pregnancy have also demonstrated the benefits of gestational diet reversals (Table 1). For example, the male offspring of obese rats given a dietary intervention during the gestational period have been found to have improved metabolic outcomes including improved insulin sensitivity both neonatally and into adulthood [33]. However, additional rodent studies highlight that a diet-reversal during pregnancy may not be sufficient to reverse the effects of maternal pre-pregnancy obesity, as observed in sheep and NHP models. For example, mouse embryos transferred at the 2-cell stage from high-fat-fed dams to control fed dams displayed poor in utero growth and neonatal catch-up growth, as well as an altered expression of imprinted genes that have been associated with obesity development suggesting that oocytes may be primed for adverse development as a direct result of poor maternal diet pre-conception [34]. These findings are supported by other rodent models that report poor liver and skeletal muscle mitochondrial health at post-natal day 35 in offspring exposed to maternal pre-pregnancy obesity [35,36]. Specifically, hepatic tissue of rat offspring born to obese dams displayed a marked decrease in the protein expression of markers of mitochondrial health and biogenesis despite both control and obese dams being fed a control diet during the gestational period [36]. 

The presence of the conflicting data between rodent and larger mammal (sheep and NHP) models may simply arise from physiological differences between these species. For example, the longer gestational period of sheep and NHP, and the fact that these species, like humans, have largely prenatal developmental processes potentially underlies the differential impacts of a gestational diet reversal intervention on fetal growth and development [37,38]. Further studies must be conducted to fully understand whether dietary changes during human pregnancy are sufficient to reverse insults from a poor maternal diet as in the NHP model and some rodent models or if human oocytes are ‘primed’ for metabolic disease with pre-gestational obesity exposure. Overall, these NHP and rodent studies demonstrate that maternal diet prior to conception and during pregnancy has a profound impact of the metabolic health of the offspring.

## 4. Maternal Dietary Fat Consumption and Offspring Metabolic Health

Human population data have suggested that circulating maternal free fatty acids levels are predictive of offspring metabolic health risks independent from measures of maternal body composition, highlighting the importance of dietary lipids during gestation [43]. Additionally, in animal-based studies, dietary fat components are altered in obese pregnancy dietary interventions further highlighting that fats themselves are important in promoting the development of metabolic disorders in exposed offspring.

Different FA species have varying impacts on metabolic health based on the length of the FA chain (short-, medium-, long or very long-chain FA) as well as on the degree of saturation of the FA [44]. For example, a diet rich in cis-monounsaturated FA species (MUFAs) and polyunsaturated fats (PUFAs) has been associated with increased levels of High-Density Lipoprotein (HDL), the “good cholesterol”, and thus a healthier lipid metabolic profile [45]. More importantly, omega-3 PUFAs have also been linked to improvements in metabolic health and function and may be an important factor in preventing insulin resistance and type 2 diabetes in obese populations [46,47]. In contrast, a high consumption of trans-unsaturated FA species has been found to lower serum levels of HDL and promote a less healthy metabolic profile [45]. Additionally, a high consumption of saturated FA species has been associated with poor metabolic profiles including increased serum levels of triglycerides, free cholesterol and low-density lipoprotein (LDL), the “bad cholesterol” [48].

More importantly, consumption of certain FA species during pregnancy has been suggested to promote the development of metabolic disorders in the offspring. For example, studies in rodent model systems have highlighted that maternal diets comprised of different saturated FA chain lengths have varying impacts on offspring later-life metabolic health [40]. Specifically, gestational diets that were overabundant in medium chain length FA species from coconut oil (55% of FA species C14:0 or shorter) resulted in decreased offspring obesity development compared to offspring exposed to a maternal overconsumption of longer-chain FA species from soybean oil (all FA C16:0 or longer) [40]. Additional rodent models have demonstrated that maternal diets rich in trans-unsaturated FA species adversely affect offspring liver mitochondrial oxidative function, as well as increase circulating levels of triglycerides, highlighting an overall dysregulation of hepatic lipid handling [39]. These studies further highlight that maternal dietary fats are an important independent factor in offspring risk for metabolic disease development. 

To determine the impact of maternal dietary fat content upon fetal health outcomes in human populations, it is important to fully understand the diet consumption patterns of pregnant women. More importantly, it is necessary to understand how these maternal diets deviate from the recommendations of government health agencies to provide insight into possible dietary interventions that can reduce offspring metabolic health complications. Canada’s food guide for example, recommends that pregnant women only consume a small amount (1–3 tbsp) of saturated fat each day. In addition to limiting saturated fat intake, it is also suggested that these less healthy FAs should be replaced with more omega-3 and -6 PUFAs. Specifically, for pregnant women, Health Canada guidelines suggest consumption of at least 200 mg of Docosahexaenoic acid (DHA) (an omega-3 PUFA), as this FA is necessary for proper fetal brain development [49]. However, despite these guidelines, analysis of dietary consumption patterns suggests that a majority of pregnant women consume diets that greatly deviate from food guide recommendations [50]. It is estimated that, on average, one-third of total caloric intake in pregnant women is from lipid sources, and while this total fat intake does not always exceed recommendations, the specific FAs that constitute total lipid intake in these women is not ideal [50,51,52]. Specifically, these women have been found to consume diets that are calorie-dense but low in nutrients, overabundant in long-chain saturated FA and lacking in important unsaturated FA species such as DHA [52,53,54].

Overall, an increased maternal consumption of saturated FA and limited intake of omega-3 PUFAs during pregnancy may be an important in utero insult that predisposes the offspring to metabolic complications early in life.

## 5. The Impact of Diet and Obesity upon the Placenta

The placenta is a transient organ composed of a heterogeneous population of cells that facilitates hormone production, fetal immunity and all gaseous, nutrient and waste transport between maternal and fetal circulation. It consists of two distinct but important populations of trophoblast cells, extravillous trophoblasts (EVTs) and villous trophoblasts that arise from the outer trophectoderm layer of the pre-implantation blastocyst. EVTs invade into the uterine wall to establish the maternofetal blood connection and anchor chorionic villi to the uterine wall, while the villous trophoblast cells of the chorionic villi act as a transport layer and comprise the barrier between maternal and fetal blood supplies. The villous trophoblast layer is comprised of two unique cell population: underlying progenitor cytotrophoblast (CT) cells and fused multi-nucleated syncytiotrophoblast (SCT) cells [55].

The CT and SCT cells of the villous trophoblast layer have been identified as the most metabolically active within the placenta, and importantly maternal gestational obesity has also been identified to negatively impact these cells [55,56,57,58,59]. Specifically, maternal obesity is often associated with increased inflammation in placental tissues highlighted by increased pro-inflammatory cytokine abundance and macrophage accumulation that can be detected as early as midgestation [41,60,61]. Additionally, maternal gestational obesity has been linked with a decreased expression of markers of mitochondrial replication, and an overall reduction in electron transport chain activity (oxidative function) leading to reduced placental ATP levels [36,56,62]. Impairments in placental functional processes are thought underlie the aberrant fetal programming that primes obesity-exposed offspring for metabolic dysfunction and ultimately metabolic disease early in life [63]. For example, NHP models have demonstrated reduced placental vascular function and increased placental inflammation with maternal obesity that can be improved with maternal diet reversal [42]. In turn, these diet reversal-induced improvements in placental function may underlie the previously observed alterations to offspring lipid and glucose metabolism [28,29,32,42]. Understanding specifically how maternal dietary fat consumption may modulate placental lipid processing functions—including lipid transport, esterification and oxidation—and what these changes mean for the developing fetus, will provide a better understanding of the mechanisms underlying early-onset metabolic disease.

In vitro cell-based analysis of the placentamay allow for such insight into the effects of maternal dietary intervention onlipid processing functions For example, CT cells have been cultured from term human placentae following planned, non-laboring Caesarian-section births and utilized to examine placental metabolic function in obese pregnancies with and without a dietary intervention [64,65]. The isolated effects of individual lipid species on placental lipid processes, independent from maternal body composition and maternal gestational diet can also be examined through the use of immortalized villous trophoblast cell lines that are available for commercial purchase. One such cell line is the BeWo cell line, which has been demonstrated as a model of placental barrier function and has been extensively utilized to examine the isolated effects that individual PUFA species have on placental lipid transport [66,67].

## 6. Regulation of Placental Lipid Transport in Obesity and the Impact of Dietary Fats

The human placenta has an extensive ability to uptake lipid species and shuttle them and their metabolic byproducts into fetal circulation. Proteomic analysis of term primary human trophoblast (PHTs) has revealed that the placenta expresses lipid transport proteins on both the apical microvillous (maternal-facing) and basolateral (fetal-facing) membranes [68]. Specifically, Fatty Acid Transport Proteins 1, 2 and 4 (FATP1, FATP2, FATP4); Fatty Acid Binding proteins 1 and 3 (FABP1, FABP3) as well as Fatty Acid Translocase (FAT/CD36) are expressed in the human placenta [68,69,70,71]. In addition, isolated PHTs have demonstrated activity of Lipoprotein Lipase (LPL) indicating that lipid species packaged as triglycerides in lipoproteins (HDL and LDL) can be processedby the placenta [72,73].

The FATPs as well as FAT/CD36 are localized on both the basolateral and apical placental membranes and are involved in transporting a wide range of FA species across the placenta [68,74]. The presence of these transporters on both membranes suggests a bidirectional transfer of NEFAs can occur to respond to the changing nutrient demands of both mother and developing fetus [68,74]. In contrast, FABP transporters that demonstrate preferential binding for PUFA species are largely localized to the maternal-facing apical membranes of the placenta [41,64]. This suggests that PUFA species are transported unidirectionally across the placenta into the fetal circulation in order to support and prioritize proper fetal brain development [68,75]. Similar to PHTs, the BeWo cell line has demonstrated the ability to uptake and transport dietary NEFAs [76]. Specifically, this cell line has been shown to express the lipid transporters: FATP1, FATP4, FAT/CD36 as well as FABP1 and FABP3 [76,77]. As BeWo cells express the same lipid transport proteins as PHTs, they may represent a viable model for studying placental barrier function and lipid transport, although caution must be taken with interpretation of data from immortalized cell lines.

**Figure 1:**
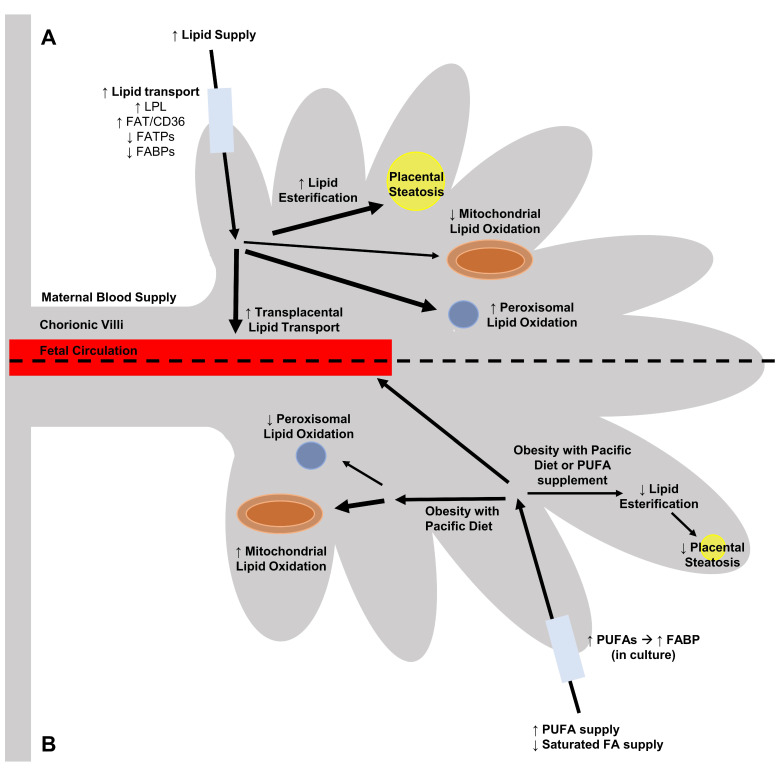
Summary description of alterations to the placental lipid processing functions of fatty acid (FA) transport, esterification and beta-oxidation under conditions of (**A**) maternal obesity and (**B**) with maternal diet improvement. Maternal gestational obesity has been associated with increased (↑) transplacental lipid transport (highlighted by increased expression of lipoprotein lipase (LPL) and fatty acid translocase (FAT/CD36) as well as decreased (↓) expression of fatty acid transport proteins (FATP) and fatty acid binding proteins (FABP)), increased placental lipid esterification and lipid droplet formation as well as decreased placental mitochondrial beta-oxidation with concomitant increased peroxisomal beta-oxidation. These changes are understood to be important in utero insults that program the development of early-life metabolic disease in the offspring from obesity-exposed pregnancies. Improved maternal diet under conditions of obesity, such as with consumption of a ‘pacific diet’ or use of dietary polyunsaturated FA (PUFA) supplements, have been associated with reduced placental steatosis and improved placental beta-oxidative function (increased mitochondrial beta-oxidation with simultaneous decreased peroxisomal beta-oxidation).

Maternal obesity during pregnancy has been associated with an altered expression and activity of the lipid transporters in the placenta. Specifically, an increase in the activity of LPL and mRNA expression of FAT/CD36 in conjunction with diminished mRNA levels of FATP1, FATP4 and FABP3 as well as reduced protein expression of FABP3 have been observed with increased maternal adiposity [72,73] (Figure 1). The observed increases in the activity and expression of placental LPL and FAT/CD36 may facilitate increased lipid transport into fetal circulation and could potentially explain the increased prevalence of LGA offspring in obese pregnancies. In contrast, the specific reduction in the expression of FATP and FABP transporters may simply reflect that the placenta is attempting to modulate lipid transportto the developing fetus under conditions of lipid overload. The notion that the placenta is able to modulate materno-fetal lipid transport in response to nutritional state is supported by recent NHP experiments that identified increased protein expression of FATP and FABP transporters under conditions of maternal nutrient restriction [78]. 

The relative influences that individual dietary FAs have on obesity-mediated altered placenta lipid transport must be understood to predict how maternal diet interventions may impact fetal metabolic disease. While almost one-third of the total lipid consumption of pregnant women is saturated fats, current research into the effects of individual NEFA supplementation on placental lipid transport has largely emphasized the effects of dietary PUFAs. Cell culture experiments conducted with the BeWo cell line have found that a 24-h exposure to 100-μM concentrations of individual unsaturated NEFAs (Oleate, DHA, and Arachidonic Acid (AA)) has no influence on placental FATP expression [76]. Similarly, there were no significant alterations in PHT FATP expression from women who took DHA supplements during the third trimester [79]. PUFAs may in contrast, have an ability to alter the expression of FABP transporters within the placenta and specifically AA has been found to increase the expression of FABP3 in BeWo cells following after 24 h in culture [77] (Figure 1). These specific increases in the expression of FABP3 in AA-treated BeWo cells may simply be reflective of the preferential transport of PUFA species by placental FABPs, [41,64]. 

Future placental research must increasingly focus on the effects of dietary saturated fats to elucidate if a maternal saturated fat overconsumption independent of body composition leads to increased materno-fetal lipid transport via LPL and FAT/CD36 mediated transport. Furthermore, understanding the molecular mechanisms that potentially regulate this increased materno-fetal lipid transport could lead to the development of pharmacological inhibitors to better modulate in utero growth.

## 7. Obesity, Diet and Placental Lipid Accumulation

The villous trophoblast cells of the placenta not only have the capability to uptake and transfer NEFAs from maternal circulation to the fetus, but also to store them as lipid droplets for future metabolic needs [80,81,82]. Analysis of the activity of FA transport proteins on placental membranes has indicated that placental lipid uptake is greater on maternal-facing membranes than on fetal-facing membranes, highlighting that placental lipid storage and/or metabolism is an important aspect of placental lipid processing [82]. More recently, CT cells were demonstrated to be the sole location of lipid esterification in cultured PHTs following treatment with fluorescent-conjugated FA derivatives [83]. This suggests that the CT cells of the villous trophoblast layer may be more important than SCT cells for lipid metabolic function in the placenta and may be a potential target of future pharmacological therapies [83].

Maternal gestational obesity has been well demonstrated to alter placental lipid storage resulting in a pathological accumulation of lipid droplets (steatosis) at term, suggesting that placental lipid droplets may be a mechanism by which the placenta modulates FA transfer to the fetus [82,84,85,86,87] (Figure 1). Analysis of the composition of these lipid droplets has demonstrated that saturated FAs and MUFAs are the predominate lipid species that are stored in obese placentae, [88]. The increase in lipid esterification and lipid droplet formation in obese placentae is potentially the result of increased formation of MUFA species via Stearoyl-CoA Desaturase (SCD-1) [85]. SCD-1 is an enzyme that is overexpressed within the obese placenta and converts the saturated FAs palmitate (16:0) and stearate (18:0) into less the lipotoxic MUFAs palmitoleate (16:1n7) and oleate (18:1n9), respectively [89]. The formation of MUFA species via SCD-1 has been previously been identified as a precursor step in the activation of WNT signaling proteins via palmitoylation [90]. More importantly, increased activity of WNT signaling proteins is involved in the pathology of placental steatosis in obesity-prone rats [91]. 

Maternal dietary supplementation with omega-3 PUFAs alone has been demonstrated to decrease placental lipid accumulation at term in obese pregnancies [86] (Figure 1). In addition, human population data have demonstrated that obese women from pacific regions such as Hawaii who naturally consume greater levels of omega-3-rich fatty foods, such as fish, have less severe placental steatosis than obese women from landlocked areas such Ohio who consume diets less plentiful in omega-3 fats [85,92] (Figure 1). These studies further highlight that maternal diet is an important regulator of placental lipid processing independent from maternal body composition. However, as previously stated, lipid esterification is also an important regulator of transplacental lipid transport. Thus, an improvement in placental steatosis with omega-3 PUFA supplements without correcting an underlying maternal overconsumption of saturated fats may be harmful to the fetus through increased transplacental lipid transport. In fact, there may be an increased risk that offspring are born LGA in pregnancies that are supplemented with omega-3 PUFA, which itself may promote the development of later life metabolic disease [93,94]. Overall, a simple dietary supplementation may not be sufficient to improve adverse fetal outcomes, and a more rigorous dietary intervention may be needed in women who overconsume saturated fats.

## 8. Diet and Placental Lipid Oxidation and Acylcarnitine Production in the Obese Environment

The dietary FA that are transported into the villous trophoblast cells from maternal circulation can additionally be metabolized via mitochondrial beta-oxidation to produce ATP necessary for the placenta to perform its biological functions. In brief, mitochondrial beta-oxidation occurs through 4 enzymatic steps in which the carbon backbone of the FA species is shortened to produce acetyl-CoA that can enter The Citric Acid Cycle. 

Immunohistological staining of isolated placental cells and western blot protein analysis of term and early gestation human placental explants has revealed that villous trophoblast cells express enzyme isoforms for all enzymatic steps in the mitochondrial beta-oxidation pathway. Both SCT and CT cells are found to express the Acyl-CoA dehydrogenase isoforms very-long-chain acyl-CoA dehydrogenase (VLCAD), long-chain acyl-CoA dehydrogenase (LCAD), and medium-chain acyl-CoA dehydrogenase (MCAD); enolyl-CoA hydratase; the 3-hydroxyacyl-CoA dehydrogenase enzyme isofroms short-chain L-3 hydroxyacyl-CoA dehydrogenase (SCHAD) and long-chain L-3 hydroxyacyl-CoA dehydrogenase (LCHAD); as well as the 3-ketoacyl-CoA thiolase enzyme isoforms long-chain 3-ketoacyl-CoA thiolase (LKAT) and short-chain 3-ketoacyl-CoA thiolase (SKAT) [95,96,97]. It is of particular interest to note that the expression levels of these beta-oxidation enzymes within placental explants is similar to that of skeletal muscle—a tissue known to be highly dependent on beta-oxidation for ATP production—highlighting that FA oxidation is critical for placental [95].Additionally, the ability of placental mitochondria to utilize lipid substrates for ATP production has been demonstrated to vary over gestation [97]. Specifically, mid-gestational placental explants display an elevated expression of mitochondrial beta-oxidation enzymes compared to term samples, indicating that the capacity of the placenta to utilize FA as a metabolic substrate diminishes as pregnancy progresses [97]. These findings suggest that the fetus may be more susceptible to influences from a maternal diet overabundant in saturated FA during late gestation when the placenta limits FA oxidation and increases trans-placental lipid transport to support rapid fetal growth.

Independently, maternal gestational obesity has been shown to impede the ability of term placental mitochondria to oxidize FA species for energy (ATP) production [85,98] (Figure 1). Observed decreases in intra-placental concentrations of acylcarnitine species (a marker of beta-oxidation) combined with an overall reduction in mitochondrial content within term obese placentae suggests that the maternal environment can negatively impact placental beta-oxidation activity [85]. However, while beta-oxidation primarily occurs within the mitochondria, placental peroxisomes have also been found to express enzymes for FA beta-oxidation [65,99,100]. Specifically, the enzymes involved in peroxisomal beta-oxidation are acyl-CoA oxidases (ACOX), D-bifunctional protein (DBP) and 3-ketoacyl-CoA thiolases [99,101]. In brief, peroxisomal beta-oxidation shortens long-chain FA species into acetyl-CoA and short-chain acyl-CoAs such as octanoyl-CoA which can then be exported into the mitochondria for complete oxidation [99,101]. More importantly, environmental cues such as fatty acid overabundance in obesity have been associated with increases in both the size and number of peroxisomes [85,102]. Additionally, maternal obesity has been linked to specific increases in the mRNA expression of peroxisomal beta-oxidation enzymes, suggesting that peroxisomal beta-oxidation is a major component of placental lipid handling in obese pregnancies [85] (Figure 1). Obese placentae were further found to have greater rates of oxidation of radio-labelled palmitate following treatment with etomoxir (a mitochondrial beta-oxidation inhibitor) than non-obese placentae highlighting that increases in peroxisomal beta-oxidation may act to modulate lipid oxidation in obese pregnancies with poor mitochondrial function [85]. Overall, these results suggest that the balance between mitochondrial and peroxisomal beta-oxidation in the placenta is disrupted by obesity. 

Maternal diet has been identified to impact placental lipid oxidative function in some obese women. Specifically, obese Hawaiian women, who consume the Pacific diet, have been found to have similar mRNA expression levels of mitochondrial and peroxisomal beta-oxidation enzymes as lean Hawaiian women [92] (Figure 1). This may suggest that the increased PUFA content of the Pacific diet could moderate the balance between mitochondrial and peroxisomal lipid oxidation. In contrast, dietary omega-3 PUFA supplementation in obese pregnancies from landlocked areas (Ohio) was not linked to alterations in mRNA expression of mitochondrial and peroxisomal beta-oxidative enzymes [86]. Additionally, omega-3 PUFA treatments did not alter [^3^H]palmitate oxidation rates in cultured villous trophoblast cells from otherwise healthy obese Ohioan women [86]. While PUFA supplementation studies have highlighted some favourable outcomes, further studies of the impact upon mitochondrial and peroxisomal beta-oxidation pathways are warranted. Furthermore, placental beta-oxidation biomarker signatures must be identified in order to appropriately monitor the effects of any dietary intervention in real time during gestation, especially in women from landlocked areas.

One potential method to quantify placental beta-oxidative function is to examine the acylcarnitine profiles of maternal blood products. Under normal physiological conditions, complete beta-oxidation occurs whereby all carbon atoms in the FA backbone are converted into acetyl-CoA molecules that are oxidized for ATP production [95,96]. However, under pathological conditions such as lipid overload, mitochondrial beta-oxidation may become incomplete resulting in accumulation of shortened chain acyl-CoA molecules within the mitochondrial matrix that may then be exported into circulation [103,104]. Analysis of differences in acylcarnitine profiles has previously been utilized to predict the presence of aberrant metabolic function in tissues including cardiac and skeletal muscle [105,106,107,108,109]. Thus, analysis of blood acylcarnitine profiles of mothers who consume poor diets throughout the gestational period may allow for the real-time identification of specific placental-derived acylcarnitine species that are predictive of aberrant placental mitochondrial beta-oxidative function. 

Acylcarnitine profiles have previously been examined as potential biomarkers for the early detection of other placental diseases such as pre-eclampsia [110,111]. Specifically, potential acylcarnitine biomarkers for the early detection of pre-eclampsia were found in both maternal serum and plasma [110,111]. In addition, acylcarnitines have also been examined as potential non-invasive biomarkers to examine placental metabolic function under conditions of maternal obesity [85,112,113]. As this field of investigation develops, it is important to note that these studies highlight that different maternal blood fractions may have differing capabilities to estimate placental metabolic function. For example, increases in some short chain acylcarnitine species are reported in maternal serum with increasing BMI [112], while no differences are found in acylcarnitine profiles in maternal plasma [113]. 

Accumulation of shortened acylcarnitine species has also previously been linked to an increased expression of pro-inflammatory molecules [104]. For example, mouse macrophage cells cultured with short-chain acylcarnitine species displayed a marked increase in the phosphorylation of the downstream effector proteins JNK and ERK which are involved in the signaling cascade of many inflammatory peptides [104]. If a maternal diet high in saturated fat can lead to incomplete placental beta-oxidation that promotes an inflammatory response, acylcarnitine analysis may be beneficial in explaining the presence of increased placental inflammation that often accompanies maternal obesity [114].

Overall, acylcarnitine analysis may represent a relatively unexplored field in placenta physiology. Analysis of differences within these profiles of obese and lean women may allow clinicians to diagnose placental mitochondrial dysfunctions in conjunction with inflammatory responses early during the gestation period. In turn, acylcarnitine biomarkers may allow clinicians to monitor the impact of dietary interventions on placental lipid handling during gestational period and modulate the course of treatment to limit the risks of offspring development of later life disease.

## 9. Conclusions

A maternal consumption of a diet high in saturated FA species and low in PUFA species during the gestational period may promote adverse placental function that underlies the development of placental and fetal metabolic dysfunction, independent to maternal body composition. Understanding the mechanisms that underlie placental metabolic dysfunctions associated with dietary fat in obese pregnancies and the accompanying offspring metabolic disorders will require a robust understanding of placental lipid transport, esterification and oxidation (Figure 1). A greater understanding of these processes will yield information that will provide frameworks from which to develop diagnostic tests to monitor the efficacy of gestational dietary interventions. Proper implementation of gestational diet improvements in obese women has the potential to limit future harm to the placenta and overall reduce risk of early-onset metabolic disease development in obesity-exposed offspring.

## Figures and Tables

**Table 1:** Summary of diet fat or feeding treatments utilized in animal models of maternal diet-induced gestational obesity and gestational high-fat exposure with and without diet reversal.

Animal Model	Dietary Fat (% Caloric Intake)	Pre-Gestational Obesity	Pre-Conception Diet Exposure	Gestational Diet Exposure	Maternal Diet Reversal	Offspring Weaning	Reference
C57/B6 mice	60% High fat diet (HFD) 25% fat control diet	HFD-induced obesity	10–12-week HFD exposure before pregnancy	HFD maintained through pregnancy and lactation	Yes—2-cell stage embryo transfer	Weaned onto control diet	Sasson [34]
C57/B6 mice	45% HFD 10% fat control diet	HFD-induced obesity	Diet commenced at 4 weeks; breeding at 10 weeks	HFD through pregnancy	No	Randomly assigned HFD or control diet	Elahi [20]
C57/BL6 mice	32% HFD 11% fat control diet	HFD-induced obesity	8-week pre-conception HFD-exposure	HFD through pregnancy	No	Fetal collections	Jones [17]
C57/B6 mice	16% HFD control diet 3% fat	Diet-induced obesity	6-week diet exposure pre-conception	HFD maintained through pregnancy and weaning	No	Pups weaned onto standard chow	Samuelsson [22]
C57/B6 mice	High trans-fat diet (6% partially hydrogenated vegetable oil + 1% soybean oil) 7% soybean oil control diet	No pre-pregnancy obesity	No HFD exposure pre-conception	High trans-fat diet through pregnancy and weaning only	No	Weaned onto control diet	de Velasco [39]
Sprague-Dawley Rats	60% HFD 24% fat control diet	HFD-induced obesity	HFD commenced Postnatal day (PND) 24; breeding PDN 120	HFD throughout pregnancy	No	Weaned onto control diet	Srinivasan [18]
Sprague-Dawley Rats	140% overfeeding model	Overfeeding-induced obesity	3-week overfeeding prior to conception	Overfeeding discontinued during pregnancy	Yes—dams switched to control feeding through pregnancy and lactation	Randomly weaned onto control (17% fat) or HFD (45% fat)	Borengasser [35]
Sprague-Dawley Rats	140% overfeeding model	Overfeeding-induced obesity	3-week overfeeding prior to conception	Overfeeding discontinued during pregnancy	Yes—dams switched to control feeding through pregnancy and lactation	Randomly weaned onto control (17% fat) or HFD (45% fat)	Borengasser [36]
Wistar Rats	45% HFD 18% fat control diet	HFD-induced obesity with pre-gestational HFD exposure	Pre-conception HFD—commenced PND 22; breeding at PND 120 Pregnancy and lactation HFD—commenced at breeding and maintained through lactation)	HFD through pregnancy	No	Randomly assigned HFD or control diet	Howie [21]
Wistar Rats	38% HFD-diets 15% fat control diet	No pre-pregnancy obesity	No HFD exposure pre-conception	HFD during pregnancy only; cross-fostered to lean dams during lactation	No	Weaned onto control diet; HFD exposure at 8 weeks	Dong [40]
Wistar Rats	20% lard supplement in HFD 5% fat control diet	HFD-induced obesity	HFD exposure from PND 21 to breeding at PND 120	HFD maintained through pregnancy and lactation	Yes—diet intervention back to control diet at PND 90	Not specified	Zambrano [33]
Sheep	155% overfeeding model	No pre-gestational obesity	Overfeeding commenced gestational day 115	Overfeeding from gestational day 115 to gestation (~day 150)	No	Control diet during lactation and weaning	Philip [26]
Sheep	150% overfeeding model	Overfeeding-induced obesity	60-day overfeeding exposure before mating	Overfeeding through gestation, control diet during lactation	No	control diet	Long [25]
Sheep	150% overfeeding model	Overfeeding-induced obesity	60-day overfeeding exposure before mating	Overfeeding until fetal collection	No	Fetal collection	Zhu [41]
Sheep	150% overfeeding model	Overfeeding-induced obesity	60-day overfeeding exposure before mating	Overfeeding continued through pregnancy (with no intervention)	Yes—150% overfeeding until gestational day 28 (with obesity intervention)	Fetal collection	Tuersunjiang [27]
Japanese Macaque	36% HFD 14% fat control diet	HFD-induced obesity	4–7-year HFD exposure pre-conception	HFD maintained through to fetal collections at gestational day 130	Yes—diet reversal 3 months prior to breeding	Fetal collection	Salati [42]
Japanese Macaque	32% HFD 14% fat control diet	HFD-induced obesity	2–4-year pre-gestational HFD induced obesity	HFD, or diet-reversal through pregnancy	Yes—pre-conception diet reversal on subsequent pregnancy	Weaned onto mothers gestational diet	McCurdy [28]
Japanese Macaque	32% HFD 14% fat control diet	HFD-induced obesity	4–7-year pre-gestational HFD exposure	HFD, or diet reversal through pregnancy	Yes—switched back to control diet in 5th breeding season	Weaned onto in utero or reverse diet	Pound [29]
Japanese Macaque	32% HFD 14% fat control diet	HFD-induced obesity	2–9-year pre-conception HFD exposure	HFD, or diet reversal through pregnancy	Yes—switched back to control diet in 9th breeding season	Fetal collections	Wesolowski [32]

## References

[1] Catalano P.M., Tyzbir E.D., Roman N.M., Amini S.B., Sims E.A. (1991). Longitudinal changes in insulin release and insulin resistance in nonobese pregnant women. Am. J. Obstet. Gynecol..

[2] Desoye G., Schweditsch M.O., Pfeiffer K.P., Zechner R., Kostner G.M. (1987). Correlation of Hormones with Lipid and Lipoprotein Levels During Normal Pregnancy and Postpartum. J. Clin. Endocrinol. Metab..

[3] Musial B., Vaughan O.R., Fernandez-Twinn D.S., Voshol P., Ozanne S.E., Fowden A.L., Sferruzzi-Perri A.N. (2017). A Western-style obesogenic diet alters maternal metabolic physiology with consequences for fetal nutrient acquisition in mice. J. Physiol..

[4] Silveira P.P., Portella A., Goldani M.Z., Barbieri A.M. (2007). Developmental origins of health and disease (DOHaD). J. Pediatr..

[5] Wadhwa P.D., Buss C., Entringer S., Swanson J.M. (2009). Developmental Origins of Health and Disease: Brief History of the Approach and Current Focus on Epigenetic Mechanisms. Semin. Reprod. Med..

[6] Forsdahl A. (1977). Are poor living conditions in childhood and adolescence an important risk factor for arteriosclerotic heart disease?. J. Epidemiol. Community Health.

[7] King J.C. (2006). Maternal Obesity, Metabolism, and Pregnancy Outcomes. Annu. Rev. Nutr..

[8] Wu G., Bazer F.W., Cudd T.A., Meininger C.J., Spencer T.E. (2004). Recent Advances in Nutritional Sciences Maternal Nutrition and Fetal. Amino Acids.

[9] (2000). Obesity: Preventing and Managing the Global Epidemic.

[10] McDonald S.D., Han Z., Mulla S., Beyene J. (2010). On behalf of the Knowledge Synthesis Group Overweight and obesity in mothers and risk of preterm birth and low birth weight infants: Systematic review and meta-analyses. BMJ.

[11] Yu Z., Han S., Zhu J., Sun X., Ji C., Guo X. (2013). Pre-Pregnancy Body Mass Index in Relation to Infant Birth Weight and Offspring Overweight/Obesity: A Systematic Review and Meta-Analysis. PLoS ONE.

[12] Da Silveira V.M.F., Horta B.L. (2008). Peso ao nascer e síndrome metabólica em adultos: Meta-análise. Revista De Saúde Pública.

[13] Boney C.M. (2005). Metabolic Syndrome in Childhood: Association with Birth Weight, Maternal Obesity, and Gestational Diabetes Mellitus. Pediatrics.

[14] Whitaker R.C. (2004). Predicting preschooler obesity at birth: The role of maternal obesity in early pregnancy. Pediatrics.

[15] Heerwagen M.J.R., Miller M.R., Barbour L.A., Friedman J.E. (2010). Maternal obesity and fetal metabolic programming: A fertile epigenetic soil. Am. J. Physiol. Integr. Comp. Physiol..

[16] Williams L., Seki Y., Vuguin P.M., Charron M.J. (2013). Animal models of in utero exposure to a high fat diet: A review. Biochim. Biophys. Acta-Bioenerg..

[17] Jones H.N., Woollett L.A., Barbour N., Prasad P.D., Powell T.L., Jansson T. (2008). High-fat diet before and during pregnancy causes marked up-regulation of placental nutrient transport and fetal overgrowth in C57/BL6 mice. FASEB J..

[18] Srinivasan M., Katewa S.D., Palaniyappan A., Pandya J.D., Patel M.S. (2006). Maternal high-fat diet consumption results in fetal malprogramming predisposing to the onset of metabolic syndrome-like phenotype in adulthood. Am. J. Physiol. Metab..

[19] Li M., Sloboda D.M., Vickers M.H. (2011). Maternal Obesity and Developmental Programming of Metabolic Disorders in Offspring: Evidence from Animal Models. Exp. Diabetes Res..

[20] Elahi M.M., Cagampang F.R., Mukhtar D., Anthony F.W., Ohri S.K., Hanson M.A. (2009). Long-term maternal high-fat feeding from weaning through pregnancy and lactation predisposes offspring to hypertension, raised plasma lipids and fatty liver in mice. Br. J. Nutr..

[21] Howie G.J., Sloboda D.M., Kamal T., Vickers M.H. (2008). Maternal nutritional history predicts obesity in adult offspring independent of postnatal diet. J. Physiol..

[22] Samuelsson A.-M., Matthews P.A., Argenton M., Christie M., McConnell J.M., Jansen E.H.M., Piersma A.H., Ozanne S.E., Fernandez-Twinn D.S., Remacle C. (2008). Diet-Induced Obesity in Female Mice Leads to Offspring Hyperphagia, Adiposity, Hypertension, and Insulin Resistance: A Novel Murine Model of Developmental Programming. Hypertension.

[23] Alberti K.G.M., Zimmet P., Shaw J.E. (2005). The metabolic syndrome—A new worldwide definition. Lancet.

[24] Rkhzay-Jaf J., O’Dowd J.F., Stocker C.J. (2012). Maternal Obesity and the Fetal Origins of the Metabolic Syndrome. Curr. Cardiovasc. Risk Rep..

[25] Long N.M., Rule D.C., Tuersunjiang N., Nathanielsz P.W., Ford S.P. (2015). Maternal Obesity in Sheep Increases Fatty Acid Synthesis, Upregulates Nutrient Transporters, and Increases Adiposity in Adult Male Offspring after a Feeding Challenge. PLoS ONE.

[26] Philp L.K., Muhlhausler B.S., Janovská A., Wittert G.A., Duffield J.A., McMillen I.C. (2008). Maternal overnutrition suppresses the phosphorylation of 5′-AMP-activated protein kinase in liver, but not skeletal muscle, in the fetal and neonatal sheep. Am. J. Physiol. Integr. Comp. Physiol..

[27] Tuersunjiang N., Odhiambo J.F., Long N.M., Shasa D.R., Nathanielsz P.W., Ford S.P. (2013). Diet reduction to requirements in obese/overfed ewes from early gestation prevents glucose/insulin dysregulation and returns fetal adiposity and organ development to control levels. Am. J. Physiol. Metab..

[28] McCurdy C.E., Bishop J.M., Williams S.M., Grayson B.E., Smith M.S., Friedman J.E., Grove K.L. (2009). Maternal high-fat diet triggers lipotoxicity in the fetal livers of nonhuman primates. J. Clin. Investig..

[29] Pound L.D., Comstock S.M., Grove K.L. (2014). Consumption of a Western-style diet during pregnancy impairs offspring islet vascularization in a Japanese macaque model. Am. J. Physiol. Metab..

[30] (2016). NCD Risk Factor Collaboration (NCD-RisC) Trends in adult body-mass index in 200 countries from 1975 to 2014: A pooled analysis of 1698 population-based measurement studies with 19·2 million participants. Lancet.

[31] (2013). Women in Canada: A Gender-Based Statistical Report (89-503-X).

[32] Wesolowski S.R., Mulligan C.M., Janssen R.C., Baker P.R., Bergman B.C., D’Alessandro A., Nemkov T., MacLean K.N., Jiang H., Dean T.A. (2018). Switching obese mothers to a healthy diet improves fetal hypoxemia, hepatic metabolites, and lipotoxicity in non-human primates. Mol. Metab..

[33] Zambrano E., Martínez-Samayoa P.M., Rodríguez-González G.L., Nathanielsz P.W. (2010). Dietary intervention prior to pregnancy reverses metabolic programming in male offspring of obese rats. J. Physiol..

[34] Sasson I.E., Vitins A.P., Mainigi M.A., Moley K.H., Simmons R.A. (2014). Pre-gestational vs gestational exposure to maternal obesity differentially programs the offspring in mice. Diabetologia.

[35] Borengasser S.J., Kang P., Faske J., Gomez-Acevedo H., Blackburn M.L., Badger T.M., Shankar K. (2014). High Fat Diet and In Utero Exposure to Maternal Obesity Disrupts Circadian Rhythm and Leads to Metabolic Programming of Liver in Rat Offspring. PLoS ONE.

[36] Borengasser S.J., Faske J., Kang P., Blackburn M.L., Badger T.M., Shankar K. (2014). In utero exposure to prepregnancy maternal obesity and postweaning high-fat diet impair regulators of mitochondrial dynamics in rat placenta and offspring. Physiol. Genom..

[37] Swanson A., David A. (2015). Animal models of fetal growth restriction: Considerations for translational medicine. Placenta.

[38] Morrison J.L., Botting K.J., Darby J.R., David A.L., Dyson R.M., Gatford K.L., Gray C., Herrera E.A., Hirst J.J., Kim B. (2018). Guinea pig models for translation of the developmental origins of health and disease hypothesis into the clinic. J. Physiol..

[39] De Velasco P.C., Chicaybam G., Ramos-Filho D.M., Dos Santos R.M.A.R., Mairink C., Sardinha F.L.C., El-Bacha T., Galina A., Tavares-Do-Carmo M.D.G. (2017). Maternal intake of trans-unsaturated or interesterified fatty acids during pregnancy and lactation modifies mitochondrial bioenergetics in the liver of adult offspring in mice. Br. J. Nutr..

[40] Dong Y.-M., Li Y., Ning H., Wang C., Liu J., Sun C. (2011). High dietary intake of medium-chain fatty acids during pregnancy in rats prevents later-life obesity in their offspring. J. Nutr. Biochem..

[41] Zhu M., Du M., Nathanielsz P.W., Ford S. (2010). Maternal obesity up-regulates inflammatory signaling pathways and enhances cytokine expression in the mid-gestation sheep placenta. Placenta.

[42] Salati J.A., Roberts V.H., Schabel M.C., Lo J.O., Kroenke C.D., Lewandowski K.S., Lindner J.R., Grove K.L., Frias A.E. (2018). Maternal high-fat diet reversal improves placental hemodynamics in a nonhuman primate model of diet-induced obesity. Int. J. Obes..

[43] Gademan M., Vermeulen M., Oostvogels A.J.J.M., Roseboom T.J., Visscher T.L.S., Van Eijsden M., Twickler M.T.B., Vrijkotte T.G.M. (2014). Maternal Prepregancy BMI and Lipid Profile during Early Pregnancy Are Independently Associated with Offspring’s Body Composition at Age 5–6 Years: The ABCD Study. PLoS ONE.

[44] Jones A.E., Stolinski M., Smith R.D., Murphy J.L., AWootton S. (1999). Effect of fatty acid chain length and saturation on the gastrointestinal handling and metabolic disposal of dietary fatty acids in women. Br. J. Nutr..

[45] Mensink R.P., Zock P., Kester A.D.M., Katan M.B. (2003). Effects of dietary fatty acids and carbohydrates on the ratio of serum total to HDL cholesterol and on serum lipids and apolipoproteins: A meta-analysis of 60 controlled trials. Am. J. Clin. Nutr..

[46] Delarue J., Le Foll C., Corporeau C., Lucas D. (2004). N-3 long chain polyunsaturated fatty acids: A nutritional tool to prevent insulin resistance associated to type 2 diabetes and obesity?. Reprod. Nutr. Dev..

[47] Lombardo Y.B., Hein G., Chicco A. (2007). Metabolic Syndrome: Effects of n-3 PUFAs on a Model of Dyslipidemia, Insulin Resistance and Adiposity. Lipids.

[48] Diniz Y.S., Cicogna A.C., Padovani C.R., Santana L.S., AFaine L., Novelli E.L. (2004). Diets rich in saturated and polyunsaturated fatty acids: Metabolic shifting and cardiac health. Nutrition.

[49] Makrides M. (2009). Is there a dietary requirement for DHA in pregnancy?. Prostaglandins Leukot. Essent. Fat. Acids.

[50] Savard C., Lemieux S., Weisnagel S.J., Fontaine-Bisson B., Gagnon C., Robitaille J., Morisset A.-S. (2018). Trimester-Specific Dietary Intakes in a Sample of French-Canadian Pregnant Women in Comparison with National Nutritional Guidelines. Nutrition.

[51] Watts V., Rockett H., Baer H.J., Leppert J., Colditz G.A. (2006). Assessing Diet Quality in a Population of Low-Income Pregnant Women: A Comparison Between Native Americans and Whites. Matern. Child. Health J..

[52] Denomme J., Stark K.D., Holub B.J. (2005). Directly Quantitated Dietary (n-3) Fatty Acid Intakes of Pregnant Canadian Women Are Lower than Current Dietary Recommendations. J. Nutr..

[53] Siega-Riz A.M., Bodnar L.M., Savitz D.A. (2002). What are pregnant women eating? Nutrient and food group differences by race. Am. J. Obstet. Gynecol..

[54] Innis S.M., Elias S.L. (2003). Intakes of essential n−6 and n−3 polyunsaturated fatty acids among pregnant Canadian women. Am. J. Clin. Nutr..

[55] Gude N., Roberts C.T., Kalionis B., King R.G. (2004). Growth and function of the normal human placenta. Thromb. Res..

[56] Mele J., Muralimanoharan S., Maloyan A., Myatt L. (2014). Impaired mitochondrial function in human placenta with increased maternal adiposity. Am. J. Physiol. Metab..

[57] Maloyan A., Mele J., Muralimanoharan S., Myatt L. (2016). Placental metabolic flexibility is affected by maternal obesity. Placenta.

[58] Kolahi K.S., Valent A.M., Thornburg K.L. (2017). Cytotrophoblast, Not Syncytiotrophoblast, Dominates Glycolysis and Oxidative Phosphorylation in Human Term Placenta. Sci. Rep..

[59] Nugent B., Bale T.L. (2015). The omniscient placenta: Metabolic and epigenetic regulation of fetal programming. Front. Neuroendocr..

[60] Roberts K., Riley S., Reynolds R., Barr S., Evans M., Statham A., Hor K., Jabbour H., Norman J., Denison F. (2011). Placental structure and inflammation in pregnancies associated with obesity. Placenta.

[61] Challier J., Basu S., Bintein T., Minium J., Hotmire K., Catalano P., Mouzon S.H.-D. (2008). Obesity in Pregnancy Stimulates Macrophage Accumulation and Inflammation in the Placenta. Placenta.

[62] Hastie R., Lappas M. (2014). The effect of pre-existing maternal obesity and diabetes on placental mitochondrial content and electron transport chain activity. Placenta.

[63] Jansson T., Powell T.L. (2007). Role of the placenta in fetal programming: Underlying mechanisms and potential interventional approaches. Clin. Sci..

[64] Li L., Schust D.J. (2015). Isolation, purification and in vitro differentiation of cytotrophoblast cells from human term placenta. Reprod. Boil. Endocrinol..

[65] Mendez-Figueroa H., Chien E.K., Ji H., Nesbitt N.L., Bharathi S.S., Goetzman E. (2012). Effects of labor on placental fatty acid β oxidation. J. Matern. Neonatal Med..

[66] Nersisyan S.A., Shkurnikov M.Y., Knyazev E.N. (2020). Factors Involved in miRNA Processing Change Its Expression Level during Imitation of Hypoxia in BeWo b30 Cells. Dokl. Biochem. Biophys..

[67] Abaidoo C., Warren M.A., Andrews P.W., Boateng K.A. (2010). A quantitative Assessment of the Morphological Characteristics of BeWo Cells as an in vitro Model of Human Trophoblast Cells. Int. J. Morphol..

[68] Campbell F.M., Bush P.G., Veerkamp J.H., Dutta-Roy A.K. (1998). Detection and cellular localization of plasma membrane-associated and cytoplasmic fatty acid-binding proteins in human placenta. Placenta.

[69] Larqué E., Demmelmair H., Klingler M., De Jonge S., Bondy B., Koletzko B. (2006). Expression pattern of fatty acid transport protein-1 (FATP-1), FATP-4 and heart-fatty acid binding protein (H-FABP) genes in human term placenta. Early Hum. Dev..

[70] Haggarty P., Ashton J., Joynson M., Abramovich D.R., Page K. (1999). Effect of Maternal Polyunsaturated Fatty Acid Concentration on Transport by the Human Placenta. Biol. Neonate.

[71] Duttaroy A.K., Basak S. (2020). Maternal dietary fatty acids and their roles in human placental development. Prostaglandins Leukot. Essent. Fat. Acids.

[72] Dubé E., Gravel A., Martin C., Desparois G., Moussa I., Ethier-Chiasson M., Forest J.-C., Giguère Y., Masse A., Lafond J. (2012). Modulation of Fatty Acid Transport and Metabolism by Maternal Obesity in the Human Full-Term Placenta1. Biol. Reprod..

[73] Segura M.T., Demmelmair H., Krauss-Etschmann S., Nathan P., Dehmel S., Padilla M.C., Rueda R., Koletzko B., Campoy C. (2017). Maternal BMI and gestational diabetes alter placental lipid transporters and fatty acid composition. Placenta.

[74] Walker N., Filis P., Soffientini U., Bellingham M., O’Shaughnessy P.J., Fowler P.A. (2017). Placental transporter localization and expression in the Human: The importance of species, sex, and gestational age differences†. Biol. Reprod..

[75] Gil-Sánchez A., Demmelmair H., Parrilla J.J., Koletzko B., Larqué E. (2011). Mechanisms involved in the selective transfer of long chain polyunsaturated fatty acids to the fetus. Front. Genet..

[76] Tobin K.A.R., Johnsen G.M., Staff A.C., Duttaroy A.K. (2009). Long-chain Polyunsaturated Fatty Acid Transport across Human Placental Choriocarcinoma (BeWo) Cells. Placenta.

[77] Leroy C., Tobin K.A.R., Basak S., Cathrine Staff A., Duttaroy A.K. (2017). Fatty acid-binding protein3 expression in BeWo cells, a human placental choriocarcinoma cell line. Prostaglandins Leukot. Essent. Fat. Acids.

[78] Chassen S.S., Ferchaud-Roucher V., Palmer C., Li C., Jansson T., Nathanielsz P.W., Powell T.L. (2020). Placental fatty acid transport across late gestation in a baboon model of intrauterine growth restriction. J. Physiol..

[79] Larqué E., Krauss-Etschmann S., Campoy C., Hartl D., Linde J., Klingler M., Demmelmair H., Caño A., Gil A., Bondy B. (2006). Docosahexaenoic acid supply in pregnancy affects placental expression of fatty acid transport proteins. Am. J. Clin. Nutr..

[80] Szabo A.J., de Lellis R., Grimaldi R.D. (1973). Triglyceride synthesis by the human placenta. Am. J. Obstet. Gynecol..

[81] Pathmaperuma A.N., Maña P., Cheung S.N., Kugathas K., Josiah A., Koina M.E., Broomfield A., Delghingaro-Augusto V., Ellwood D.A., Dahlstrom J.E. (2010). Fatty acids alter glycerolipid metabolism and induce lipid droplet formation, syncytialisation and cytokine production in human trophoblasts with minimal glucose effect or interaction. Placenta.

[82] Perazzolo S., Hirschmugl B., Wadsack C., Desoye G., Lewis R.M., Sengers B.G. (2017). The influence of placental metabolism on fatty acid transfer to the fetus. J. Lipid Res..

[83] Kolahi K., Louey S., Varlamov O., Thornburg K. (2016). Real-time tracking of BODIPY-C12 long-chain fatty acid in human term placenta reveals unique lipid dynamics in cytotrophoblast cells. PLoS ONE.

[84] Margariti E., Deutsch M., Manolakopoulos S., Kaflri G., Tiniakos D., Papatheodoridis G.V. (2011). Non-alcoholic fatty liver disease (nafld) may develop in patients with normal body mass index (BMI). J. Hepatol..

[85] Calabuig-Navarro V., Haghiac M., Minium J., Glazebrook P., Ranasinghe G.C., Hoppel C., Hauguel de-Mouzon S., Catalano P., O’Tierney-Ginn P. (2017). Effect of Maternal Obesity on Placental Lipid Metabolism. Endocrinol..

[86] Calabuig-Navarro V., Puchowicz M., Glazebrook P., Haghiac M., Minium J., Catalano P., Hauguel de Mouzon S., O’Tierney-Ginn P. (2016). Effect of ω-3 supplementation on placental lipid metabolism in overweight and obese women. Am. J. Clin. Nutr..

[87] Cetin I., Parisi F., Berti C., Mandò C., Desoye G. (2012). Placental fatty acid transport in maternal obesity. J. Dev. Orig. Health Dis..

[88] Gázquez A., Uhl O., Ruíz-Palacios M., Gill C., Patel N., Koletzko B., Poston L., Larqué E. (2018). Placental lipid droplet composition: Effect of a lifestyle intervention (UPBEAT) in obese pregnant women. Biochim. Biophys. Acta-Mol. Cell Biol. Lipids.

[89] Yang C., Lim W., Bazer F.W., Song G. (2018). Down-regulation of stearoyl-CoA desaturase-1 increases susceptibility to palmitic-acid-induced lipotoxicity in human trophoblast cells. J. Nutr. Biochem..

[90] Rios-Esteves J., Resh M.D. (2013). Stearoyl CoA Desaturase Is Required to Produce Active, Lipid-Modified Wnt Proteins. Cell Rep..

[91] Strakovsky R.S., Pan Y.-X. (2012). A Decrease in DKK1, a WNT Inhibitor, Contributes to Placental Lipid Accumulation in an Obesity-Prone Rat Model1. Biol. Reprod..

[92] Alvarado F.L., Calabuig-Navarro V., Haghiac M., Puchowicz M., Tsai P.-J.S., O’Tierney-Ginn P. (2018). Maternal obesity is not associated with placental lipid accumulation in women with high omega-3 fatty acid levels. Placenta.

[93] Middleton P., Gomersall J.C., Gould J.F., Shepherd E., Olsen S.F., Makrides M. (2018). Omega-3 fatty acid addition during pregnancy. Cochrane Database Syst. Rev..

[94] Vinding R.K., Stokholm J., Sevelsted A., Chawes B.L., Bønnelykke K., Barman M., Jacobsson B., Bisgaard H. (2019). Fish Oil Supplementation in Pregnancy Increases Gestational Age, Size for Gestational Age, and Birth Weight in Infants: A Randomized Controlled Trial. J. Nutr..

[95] Shekhawat P., Bennett M.J., Sadovsky Y., Nelson D.M., Rakheja D., Strauss A.W. (2003). Human placenta metabolizes fatty acids: Implications for fetal fatty acid oxidation disorders and maternal liver diseases. Am. J. Physiol. Endocrinol. Metab..

[96] Oey N.A., Den Boer M.E.J., Ruiter J.P.N., Wanders R.J.A., Wanders R.J.A., Duran M., Waterham H.R., Boer K., van der Post J.A.M., Wijburg F.A. (2003). High activity of fatty acid oxidation enzymes in human placenta: Implications for fetal-maternal disease. J. Inherit. Metab. Dis..

[97] Rakheja D., Bennett M.J., Foster B.M., Domiati-Saad R., Rogers B.B. (2002). Evidence for Fatty Acid Oxidation in Human Placenta, and the Relationship of Fatty Acid Oxidation Enzyme Activities with Gestational Age. Placenta.

[98] Boyle K.E., Patinkin Z.W., Shapiro A.L.B., Bader C., Vanderlinden L., Kechris K., Janssen R.C., Ford R.J., Smith B.K., Steinberg G.R. (2017). Maternal obesity alters fatty acid oxidation, AMPK activity, and associated DNA methylation in mesenchymal stem cells from human infants. Mol. Metab..

[99] Wanders R.J.A., Waterham H.R., Ferdinandusse S. (2016). Metabolic Interplay between Peroxisomes and Other Subcellular Organelles Including Mitochondria and the Endoplasmic Reticulum. Front. Cell Dev. Biol..

[100] Hashimoto T. (2000). Peroxisomal beta-oxidation enzymes. Cell Biochem. Biophys..

[101] Van Veldhoven P.P. (2010). Biochemistry and genetics of inherited disorders of peroxisomal fatty acid metabolism. J. Lipid Res..

[102] Huang T.-Y., Zheng D., Hickner R.C., Brault J.J., Cortright R.N. (2019). Peroxisomal gene and protein expression increase in response to a high-lipid challenge in human skeletal muscle. Metabolism.

[103] Moore K.H., Radloff J.F., Hull F.E., Sweeley C.C. (1980). Incomplete fatty acid oxidation by ischemic heart: Beta-hydroxy fatty acid production. Am. J. Physiol..

[104] Rutkowsky J.M., Knotts T.A., Ono-Moore K.D., McCoin C.S., Huang S., Schneider D., Singh S., Adams S.H., Hwang D.H. (2014). Acylcarnitines activate proinflammatory signaling pathways. Am. J. Physiol. Endocrinol. Metab..

[105] Koves T.R., Ussher J.R., Noland R.C., Slentz D., Mosedale M., Ilkayeva O., Bain J., Stevens R., Dyck J.R.B., Newgard C.B. (2008). Mitochondrial Overload and Incomplete Fatty Acid Oxidation Contribute to Skeletal Muscle Insulin Resistance. Cell Metab..

[106] Baker P.R., Boyle K.E., Koves T.R., Ilkayeva O.R., Muoio D.M., Houmard J.A., Friedman J.E. (2015). Metabolomic analysis reveals altered skeletal muscle amino acid and fatty acid handling in obese humans. Obesity.

[107] Baker P.R., Patinkin Z., Shapiro A.L., De La Houssaye B.A., Woontner M., Boyle K.E., Vanderlinden L., Dabelea D., Friedman J.E. (2017). Maternal obesity and increased neonatal adiposity correspond with altered infant mesenchymal stem cell metabolism. JCI Insight.

[108] Ruiz M., Labarthe F., Fortier A., Bouchard B., Thompson Legault J., Bolduc V., Rigal O., Chen J., Ducharme A., Crawford P.A. (2017). Circulating acylcarnitine profile in human heart failure: A surrogate of fatty acid metabolic dysregulation in mitochondria and beyond. Am. J. Physiol. Heart Circ. Physiol..

[109] Turer A.T., Stevens R.D., Bain J.R., Muehlbauer M.J., van der Westhuizen J., Mathew J.P., Schwinn D.A., Glower D.D., Newgard C.B., Podgoreanu M.V. (2009). Metabolomic profiling reveals distinct patterns of myocardial substrate use in humans with coronary artery disease or left ventricular dysfunction during surgical ischemia/reperfusion. Circulation.

[110] Thiele I.G.I., Niezen-Koning K.E., van Gennip A.H., Aarnoudse J.G. (2004). Increased Plasma Carnitine Concentrations in Preeclampsia. Obstet. Gynecol..

[111] Koster M.P.H., Vreeken R.J., Harms A.C., Dane A.D., Kuc S., Schielen P.C.J.I., Hankemeier T., Berger R., Visser G.H.A., Pennings J.L.A. (2015). First-Trimester Serum Acylcarnitine Levels to Predict Preeclampsia: A Metabolomics Approach. Dis. Markers.

[112] Ryckman K., Donovan B., Fleener D., Bedell B., Borowski K. (2016). Pregnancy-Related Changes of Amino Acid and Acylcarnitine Concentrations: The Impact of Obesity. Am. J. Perinatol. Rep..

[113] Hellmuth C., Lindsay K.L., Uhl O., Buss C., Wadhwa P.D., Koletzko B., Entringer S. (2017). Association of maternal prepregnancy BMI with metabolomic profile across gestation. Int. J. Obes..

[114] Sampey B.P., Freemerman A.J., Zhang J., Kuan P.-F., Galanko J.A., O’Connell T.M., Ilkayeva O.R., Muehlbauer M.J., Stevens R.D., Newgard C.B. (2012). Metabolomic Profiling Reveals Mitochondrial-Derived Lipid Biomarkers That Drive Obesity-Associated Inflammation. PLoS ONE.

